# Association of HCMV IE1 protein and UL123 DNA with clinicopathological features and survival in patients with glioma

**DOI:** 10.3389/fcimb.2026.1975136

**Published:** 2026-10-20

**Authors:** Zheng Hao, Qi Liu, Yanzhuo Guo, Junjie Zhang, Rui Ding, Laizhao Chen, Xiaofeng Yin

**Affiliations:** Department of Neurosurgery, Second Hospital of Shanxi Medical University, Taiyuan, China

**Keywords:** glioma, hcmv, immediate-early 1 protein, overall survival, prognosis

## Abstract

**Background:**

The reported presence of human cytomegalovirus (HCMV) in glioma varies across studies, partly because protein- and DNA-based assays may yield different results. This analysis examined the association of HCMV immediate-early 1 (IE1) protein and UL123 DNA markers and their associations with glioma features and survival.

**Methods:**

A dataset containing 221 paired glioma records was analyzed. IE1 status was represented as an immunohistochemistry result, and UL123 DNA status was represented as a quantitative real-time PCR result. Associations with clinicopathological characteristics were examined using Pearson’s chi-square tests. Agreement between IE1 IHC and UL123 qPCR was evaluated using Cohen’s kappa and McNemar’s test. Overall survival was analyzed using Kaplan–Meier estimates and two-sided log-rank tests without multivariable adjustment.

**Results:**

IE1 high status was present in 135 records (61.1%), and UL123 positive status was present in 124 (56.1%). The assays agreed in 162 records (73.3%; kappa = 0.451), and their positive rates did not differ significantly (McNemar P = 0.193). Both markers were more frequent in higher-grade, IDH1 R132H-negative, older, and lower-KPS groups. The 36-month overall survival rate was 25.9% for IE1-high records and 54.7% for IE1-low records. Corresponding rates were 25.0% and 52.6% for UL123-positive and UL123-negative records. Double-positive records had lower 36-month overall survival than double-negative records (21.0% vs. 59.7%; log-rank P < 0.001).

**Conclusion:**

IE1 protein and UL123 DNA showed agreements and were associated with adverse clinicopathological features and shorter overall survival. These findings support further validation of standardized HCMV testing in independent glioma cohorts.

## 1 Introduction

Adult gliomas are a diverse group of brain tumors. Their diagnosis is based on tissue features and molecular changes, including isocitrate dehydrogenase (IDH) status (Louis et al., 2021). These criteria have improved tumor classification. However, high-grade gliomas remain difficult to treat. Standard treatment for glioblastoma includes maximal safe surgery, radiotherapy, and temozolomide (Stupp et al., 2005). Tumor recurrence is common, and survival remains poor. Outcomes also differ among patients with tumors that appear similar. Other factors may therefore be linked to tumor progression and patient survival.

Human cytomegalovirus (HCMV) has been studied as one possible factor. In 2002, HCMV DNA and proteins were reported in malignant gliomas but not in nearby normal brain tissue (Cobbs et al., 2002). Later studies also detected HCMV immediate-early proteins and viral DNA in glioblastoma and other gliomas (Mitchell et al., 2008; Scheurer et al., 2008). These findings led researchers to suggest that HCMV may affect tumor behavior without directly causing the tumor (Dziurzynski et al., 2012). The HCMV UL123 gene encodes immediate-early 1 (IE1) protein. IE1 helps control viral gene expression and the host antiviral response (Paulus and Nevels, 2009). Testing both IE1 protein and UL123 DNA may provide more complete information about HCMV in glioma tissue.

However, studies have reported different results. Some studies detected HCMV in many glioma samples. Other studies used quantitative real-time PCR, immunohistochemistry, and *in situ* hybridization but did not detect HCMV in glioblastoma (Yamashita et al., 2014; Holdhoff et al., 2017). A systematic review found that HCMV proteins were detected more often than viral DNA or RNA (Peredo-Harvey et al., 2021). Detection rates also differed by test method and tissue protocol (Peredo-Harvey et al., 2021). Several factors may affect the results. These include low viral DNA levels, uneven distribution within the tumor, tissue storage, antibody specificity, and test cutoffs (Mitchell et al., 2008; Yamashita et al., 2014; Holdhoff et al., 2017; Peredo-Harvey et al., 2021). Results from a single method may therefore be difficult to compare across studies.

The relation between HCMV markers and patient outcome is also unclear. One study found that higher HCMV immediate-early protein levels in tumor tissue were associated with shorter survival in patients with glioblastoma (Rahbar et al., 2013). However, differences in HCMV detection methods make this association difficult to confirm. To address this gap, studies should test HCMV protein and DNA in the same tissue samples while considering tumor grade, molecular features, and patient outcome. In this study, we measured HCMV IE1 protein by immunohistochemistry and UL123 DNA by quantitative real-time PCR in 221 glioma samples. We assessed agreement between the assays and examined their relationships with clinical features, tumor characteristics, and overall survival. By evaluating IE1 protein and UL123 DNA in the same cohort, this study may clarify whether combined HCMV testing is associated with aggressive glioma features. The findings may provide a guide for future studies evaluating standardized HCMV assays and the potential prognostic relevance of these markers in glioma.

## 2 Materials and methods

### 2.1 Study design and patients

This study included 221 patients with histologically confirmed glioma from the second hospital of Shanxi Medical University between March 2018 and December 2022. No patients underwent radiation or chemotherapy prior to surgical therapy. The retrospective use of tumor specimens and clinical data was approved by the Ethics Committee of the Second Hospital of Shanxi Medical University on 2024(approval no. [2024] YX_No.308).

Clinical data included age, sex, WHO tumor grade, IDH1 R132H status, preoperative Karnofsky Performance Status (KPS), and survival information. Age was grouped as younger than 50 years or 50 years and older. Preoperative KPS scores were grouped as below 80 or 80 and above. WHO grades I–II were classified as low-grade glioma, whereas grades III–IV were classified as high-grade glioma.

Overall survival (OS) was calculated from date of surgery until death from any cause or the last follow-up. Patients who remained alive were censored at the date of their last confirmed follow-up. Survival status and estimated survival probabilities were assessed at 36 months. Postoperative treatment and extent of resection were not included in the survival analyses.

### 2.2 Ethics approval

The study protocol was approved by the Ethics Committee of Second Hospital of Shanxi Medical University (approval number: [2024] YX_No.308). Tumor specimens were obtained during surgical resection or biopsy and were processed as paraffin-embedded tissue. Histological diagnosis and tumor grade were reviewed by 3 pathologists according to the WHO classification of central nervous system tumors.

### 2.3 Immunohistochemical detection of HCMV IE1 protein

HCMV IE1 protein was examined by immunohistochemistry (IHC) in tumor specimens from all 221 patients. Tissue sections measuring 4μm were prepared. Sections were processed using deparaffinization and rehydration procedure and underwent antigen retrieval. Sections were incubated with a mouse monoclonal antibody against HCMV IE1 (Abcam, ab53495 number, 10 μg/ml). Nonimmune mouse IgG was used in place of the primary antibody as a negative control.

IE1 staining intensity was scored as 0 (no staining), 1 (weak staining), 2 (moderate staining), or 3 (strong staining). The proportion of stained tumor cells was scored as 1 for 1–25%, 2 for 26–50%, 3 for 51–75%, and 4 for 76–100%. Specimens without stained tumor cells received a final score of 0. The final staining score was calculated by multiplying the intensity score by the proportion score, resulting in a possible score of 0–12. Tumors with a final score of ≥4 were classified as IE1-high, whereas tumors with a score of <4 were classified as IE1-low. The stained sections were independently evaluated by 3 pathologists who were blinded to the qPCR findings and clinical outcomes. Disagreements were resolved by joint review.

### 2.4 Detection of HCMV UL123 DNA by quantitative real-time PCR

HCMV UL123 DNA was assessed by quantitative real-time PCR (qPCR). Each tumor DNA sample was tested in three technical replicates. The mean Ct was calculated as the mean of the three replicate Ct values. A mean Ct of 35 was specified as an operational classification threshold. Samples with a mean Ct <35 were classified as UL123-positive, whereas samples with a mean Ct ≥35 were classified as UL123-negative. The primer sequences used for UL123 amplification were as follows: forward, 5′-TGACGCTTGTATGATGACCATGTAC-3′; reverse, 5′-CAGCATCACACTAGTCTCCTCTAAG-3′. Amplification specificity was assessed using melting-curve analysis. The available assay documentation does not establish sample-specific DNA amplifiability in UL123-negative specimens. These classifications therefore do not exclude low viral DNA levels, DNA degradation, or PCR inhibition.

### 2.5 Comparison of IE1 protein and UL123 DNA results

IHC and qPCR results were paired for each tumor. Samples with IE1-high staining and UL123-positive according to the Ct threshold were classified as double-positive. Samples with IE1-low staining and UL123-negative according to the Ct threshold were classified as double-negative. The remaining samples were classified as discordant.

Overall agreement was calculated as the number of double-positive and double-negative samples divided by the total number of paired samples. Agreement was also examined separately by WHO grade, age group, and sex. Patients with discordant results were excluded only from analyses comparing the survival of double-positive and double-negative groups.

### 2.6 Statistical analysis

Categorical variables are presented as numbers and percentages. Associations between HCMV marker status and clinicopathological characteristics were assessed using two-sided Pearson χ² tests. The patient was the independent unit for all analyses.

The association between IHC and qPCR results was assessed using Pearson’s χ² test and the phi coefficient. Overall agreement was reported as a percentage, and Cohen’s kappa was used to estimate agreement beyond chance. McNemar’s test was used to compare paired positive rates between IHC and qPCR.

OS was estimated using the Kaplan–Meier method, and survival curves were compared using two-sided log-rank tests. Median OS and 36-month OS probabilities were reported for each marker group. Survival analyses were repeated within WHO grade I–II and grade III–IV groups.

All statistical analyses were performed using SPSS. All tests were two-sided, and P < 0.05 was considered statistically significant.

## 3 Results

### 3.1 Clinicopathological characteristics and HCMV IE1 protein expression in glioma

A total of 221 patients were enrolled in this study, among whom 86 (38.9%) exhibited low or negative IE1 expression (IE1-low group) and 135 (61.1%) showed high or positive IE1 expression (IE1-high group). The baseline clinical and pathological characteristics of the study cohort are detailed in **Table 1**.

**Table 1:** Clinicopathological characteristics of 221 glioma patients and correlation with HCMV IE1 expression.

Characteristic	Total	IE1-Low	IE1-High	χ²	p-value
Age (years)					
<50	84	48	36	18.942	<0.001
>=50	137	38	99		
Sex					
Male	131	53	78	0.323	0.570
Female	90	33	57		
WHO grade					
Grade I/II (LGG)	70	48	22	37.908	<0.001
Grade III/IV (HGG)	151	38	113		
IDH1 status					
Mutant (R132H)	83	59	24	57.870	<0.001
Wild-type	138	27	111		
Pre-operative KPS score					
<80	101	31	70	5.288	0.021
>=80	120	55	65		

Data are numbers of patients unless otherwise indicated. P values were calculated using two-sided Pearson χ² tests. HGG, high-grade glioma; IDH1, isocitrate dehydrogenase 1; KPS, Karnofsky Performance Status; LGG, low-grade glioma. A P value <0.05 was considered statistically significant.

The high IE1 expression was strongly associated with more aggressive histopathological and molecular features. Specifically, IE1-high status was more frequent in grade III–IV records than in grade I–II records (74.8% versus 31.4%; P < 0.001). Genetically, it was also more frequent in IDH1 R132H-negative records than in mutant records (80.4% versus 28.9%; P < 0.001).

No statistically significant difference was observed between the IE1-low and IE1-high groups regarding patient sex. However, IE1 expression status was significantly correlated with age, which IE1 positivity was higher in patients aged ≥50 years than in those aged <50 years (72.3% vs. 42.9%). In terms of functional status, patients with IE1-high tumors were more likely to have a preoperative KPS score below 80 than patients with IE1-low tumors (51.9% vs. 36.0%; χ² = 5.288, P = 0.021).

### 3.2 Immunohistochemical detection of HCMV IE1 expression in glioma tissues

Immunohistochemistry was performed to examine HCMV IE1 protein expression in tissues from 221 patients with glioma. IE1 immunoreactivity was observed in glioma cells and was present in both the nuclei and cytoplasm of positive tumor cells. Nuclear staining was relatively clear, while the intensity and distribution of cytoplasmic staining varied among tumor cells. According to the staining intensity and percentage of positive cells, 135 of the 221 glioma samples (61.1%) were classified as having high IE1 expression, while 86 samples (38.9%) showed low or negative expression.

IE1 expression differed significantly between low- and high-grade gliomas. Among the 70 low-grade gliomas (WHO grades I–II), 22 cases (31.4%) showed high IE1 expression, whereas 48 cases (68.6%) showed low expression. By comparison, high IE1 expression was detected in 113 of the 151 high-grade gliomas (WHO grades III–IV; 74.8%), while only 38 cases (25.2%) showed low expression (**Table 1**). The difference between the two groups was statistically significant (P < 0.001). In low-grade gliomas, IE1 staining was generally weak and limited to scattered glioma cells. In high-grade gliomas, positive cells were more widely distributed, and both nuclear and cytoplasmic staining were more obvious (**Figure 1**). Isotype control (non-immune mouse IgG) was used in place of the primary antibody as a negative control, demonstrating the absence of non-specific background staining. These results indicate that HCMV IE1 expression increases with glioma grade.

**Figure 1:**
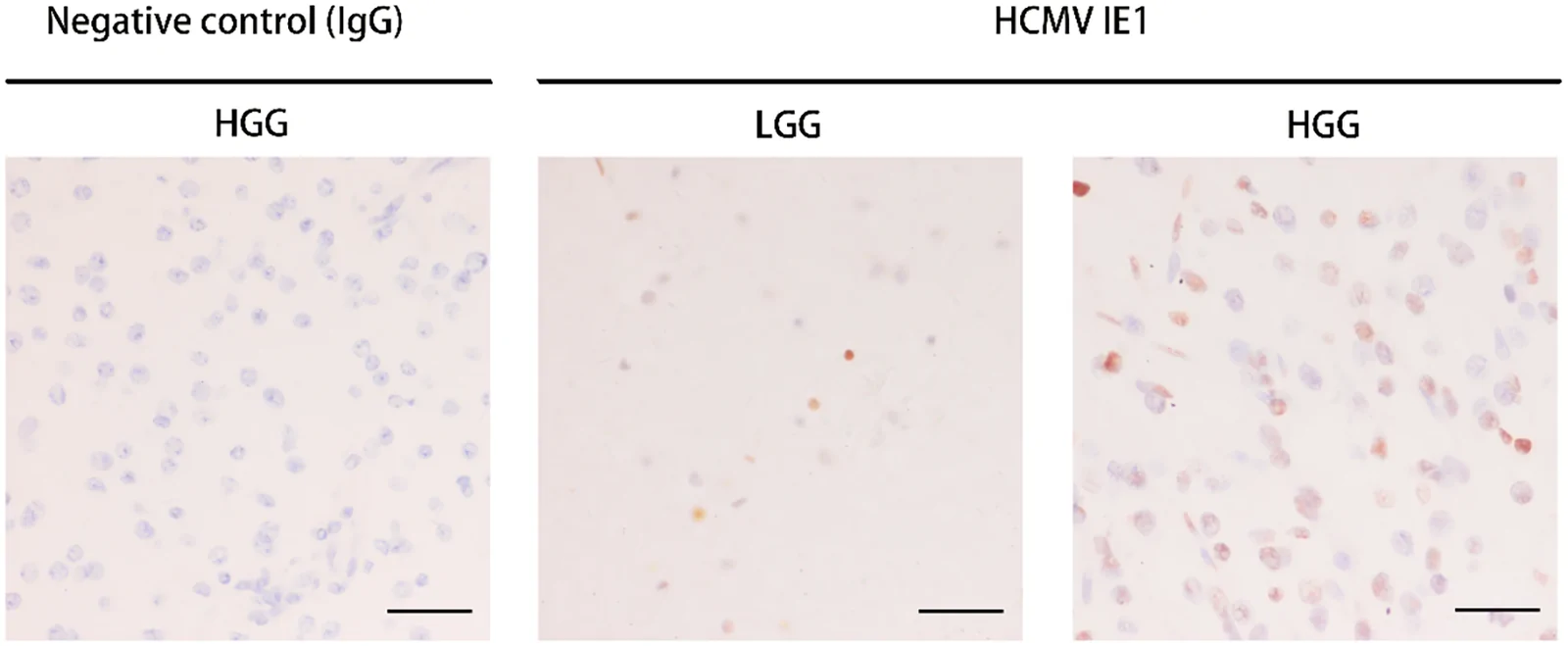
Representative immunohistochemical detection of HCMV IE1 protein in glioma tissues. The left panel shows a high-grade glioma incubated with mouse IgG instead of the primary antibody. The middle and right panels show representative IE1 staining in low-grade and high-grade gliomas, respectively. IE1 immunoreactivity was sparse and weak in low-grade glioma but more widespread in high-grade glioma, with nuclear and cytoplasmic staining. LGG, low-grade glioma; HGG, high-grade glioma. Total magnification, ×400; scale bars, 50 μm.

### 3.3 Quantitative real-time PCR detection of HCMV *UL123* in glioma tissues

Quantitative real-time PCR was performed to detect HCMV *UL123*, which encodes the IE1 protein, in glioma tissues from 221 patients. Positive *UL123* signals were detected in 124 samples (56.1%), while 97 samples (43.9%) were classified as negative. The distribution differed markedly according to tumor grade. Only 18 of 70 WHO grade I–II gliomas (25.7%) showed positive results, compared with 106 of 151 WHO grade III–IV gliomas (70.2%; χ²=38.432, P<0.001). Thus, the qPCR assay showed the same grade-related increase observed by immunohistochemistry, although the qPCR-positive proportions were lower and were not identical to the corresponding IE1 protein results (**Table 2**).

**Table 2:** Clinicopathological characteristics of 221 patients with glioma according to HCMV UL123 qPCR result.

Characteristic	Category	Total	Negative n (%)	Positive n (%)	χ²	P value
Age (years)	<50	84	53 (63.1)	31 (36.9)	20.292	**<0.001**
	>=50	137	44 (32.1)	93 (67.9)		
Sex	Male	131	58 (44.3)	73 (55.7)	0.019	0.890
	Female	90	39 (43.3)	51 (56.7)		
WHO tumor grade	Grade I/II (LGG)	70	52 (74.3)	18 (25.7)	38.432	**<0.001**
	Grade III/IV (HGG)	151	45 (29.8)	106 (70.2)		
IDH1 mutation status	Mutant (R132H)	83	61 (73.5)	22 (26.5)	47.298	**<0.001**
	Wild-type	138	36 (26.1)	102 (73.9)		
KPS score	<80	101	37 (36.6)	64 (63.4)	3.979	**0.046**
	>=80	120	60 (50.0)	60 (50.0)		
3-year overall survival	Alive	82	51 (62.2)	31 (37.8)	17.736	**<0.001**
	Deceased	139	46 (33.1)	93 (66.9)		

P values were calculated using two-sided Pearson χ² tests. HGG, high-grade glioma; IDH1, isocitrate dehydrogenase 1; KPS, Karnofsky Performance Status; LGG, low-grade glioma; qPCR, quantitative real-time polymerase chain reaction.

HCMV *UL123* positivity was also associated with age. Positive signals were found in 31 of 84 patients aged <50 years (36.9%) and in 93 of 137 patients aged ≥50 years (67.9%; χ²=20.292, P<0.001). In contrast, no significant difference was identified between male and female patients. The corresponding high rates were 55.7% (73/131) and 56.7% (51/90), respectively (χ²=0.019, P = 0.890).

The *UL123* qPCR result was strongly related to IDH1 mutation status. Positive *UL123* signals were present in 22 of 83 IDH1 R132H-mutant tumors (26.5%) but in 102 of 138 IDH1 R132H-negative tumors (73.9%; χ²=47.298, P<0.001). Patients with a preoperative KPS score <80 also had a higher positive rate than those with a score ≥80 (63.4% vs. 50.0%; χ²=3.979, P = 0.046). Overall, increased HCMV *UL123* detection was associated with higher tumor grade, older age, IDH1 wild-type status, poorer preoperative performance whereas no association was observed with sex.

### 3.4 Correlation between HCMV IE1 protein expression by immunohistochemistry and HCMV UL123 detection by quantitative real-time PCR

To determine whether IE1 protein expression was related to the presence of the corresponding HCMV gene in glioma tissue, the immunohistochemical findings were compared with qPCR detection of *UL123* in the same 221 samples. High IE1 staining was found in 135 tumors (61.1%), whereas 124 (56.1%) were classified as high for *UL123* by qPCR. The two tests gave the same result in 162 cases, including 100 positive and 62 negative samples, for an overall agreement of 73.3%. Cohen’s kappa was 0.451, and IE1 staining was positively correlated with *UL123* detection (phi=0.454, P<0.001). Among the 59 cases with different results, 35 were positive by IHC but negative by qPCR, and 24 were negative by IHC but positive by qPCR. The difference between the paired detection rates was small and was not statistically significant (McNemar chi-square=1.695, P = 0.193) (**Table 3**).

**Table 3:** Association between HCMV IE1 protein and HCMV *UL123* gene in 221 glioma specimens.

Category	N	IHC + (%)	qPCR + (%)	Both +	Both _	IHC+/ qPCR-	IHC-/ qPCR+	Agreement (%)	Cohen’s kappa	Phi	Association P value	McNemar P value
All patients	221	135 (61.1)	124 (56.1)	100	62	35	24	73.3	0.451	0.454	<0.001	0.193
Grade I/II (LGG)	70	22 (31.4)	18 (25.7)	11	41	11	7	74.3	0.373	0.376	0.002	0.480
Grade III/IV (HGG)	151	113 (74.8)	106 (70.2)	89	21	24	17	72.8	0.321	0.323	<0.001	0.349
<50	84	36 (42.9)	31 (36.9)	21	38	15	10	70.2	0.382	0.385	<0.001	0.424
>=50	137	99 (72.3)	93 (67.9)	79	24	20	14	75.2	0.410	0.412	<0.001	0.391
Male	131	78 (59.5)	73 (55.7)	56	36	22	17	70.2	0.391	0.392	<0.001	0.522
Female	90	57 (63.3)	51 (56.7)	44	26	13	7	77.8	0.539	0.544	<0.001	0.264

Association P values test the association between the two assays; McNemar P values compare paired positive rates. IHC, immunohistochemistry; qPCR, quantitative real-time polymerase chain reaction.

When tumors were grouped by WHO grade, the association between the two tests remained significant. In grade I/II gliomas, 22 of 70 samples (31.4%) showed high IE1 staining and 18 (25.7%) showed high *UL123* detection. Both tests were positive in 11 cases and negative in 41, giving an agreement of 74.3% (kappa=0.373; phi=0.376; P = 0.002). In grade III/IV gliomas, high findings were obtained in 113 of 151 samples (74.8%) by IHC and in 106 (70.2%) by qPCR. A total of 89 samples were positive and 21 were negative by both tests, with an agreement of 72.8% (kappa=0.321; phi=0.323; P<0.001). The positive rates did not differ significantly between the two methods in either the grade I/II group (McNemar P = 0.480) or the grade III/IV group (P = 0.349).

A significant correlation was also found in both age groups. Among patients aged <50 years, 36 of 84 tumors (42.9%) were high by IHC and 31 (36.9%) by qPCR. Both methods were positive in 21 cases and negative in 38, resulting in an agreement of 70.2% (kappa=0.382; phi=0.385; P<0.001). In patients aged 50 years or older, positive results were obtained in 99 of 137 tumors (72.3%) by IHC and 93 (67.9%) by qPCR. The results were the same in 103 cases, including 79 positive and 24 negative tumors, for an agreement of 75.2% (kappa=0.410; phi=0.412; P<0.001). No significant difference in the paired positive rates was observed in the younger or older group (McNemar P = 0.424 and P = 0.391, respectively).

Similar findings were observed after stratification by sex. In male patients, 78 of 131 tumors (59.5%) were high for IE1 by IHC and 73 (55.7%) were positive for *UL123* by qPCR. The two tests gave the same result in 92 tumors, corresponding to an agreement of 70.2% (kappa=0.391; phi=0.392; P<0.001). In female patients, the respective positive rates were 63.3% (57/90) and 56.7% (51/90). The results agreed in 70 tumors, and the agreement rate was 77.8% (kappa=0.539; phi=0.544; P<0.001). McNemar testing showed no significant difference between the methods in males (P = 0.522) or females (P = 0.264). Thus, the IHC and qPCR results were significantly correlated in every grade, age, and sex group, although some tumors gave different results with the two methods.

### 3.5 Association of HCMV IE1 protein and UL123 gene status with glioma patients survival

To determine whether the presence of HCMV IE1 protein or *UL123* DNA was associated with glioma patients’ survival, overall survival was analyzed using the Kaplan-Meier method. Patients with IE1-high tumors had significantly shorter overall survival than those with IE1-low tumors (P<0.001; **Figure 2A**). The 36-month overall survival rates were 25.9% and 54.7%, respectively. Median overall survival was 18.2 months in the IE1-high group, whereas the median was not reached in the IE1-low group (**Table 4**).

**Figure 2:**
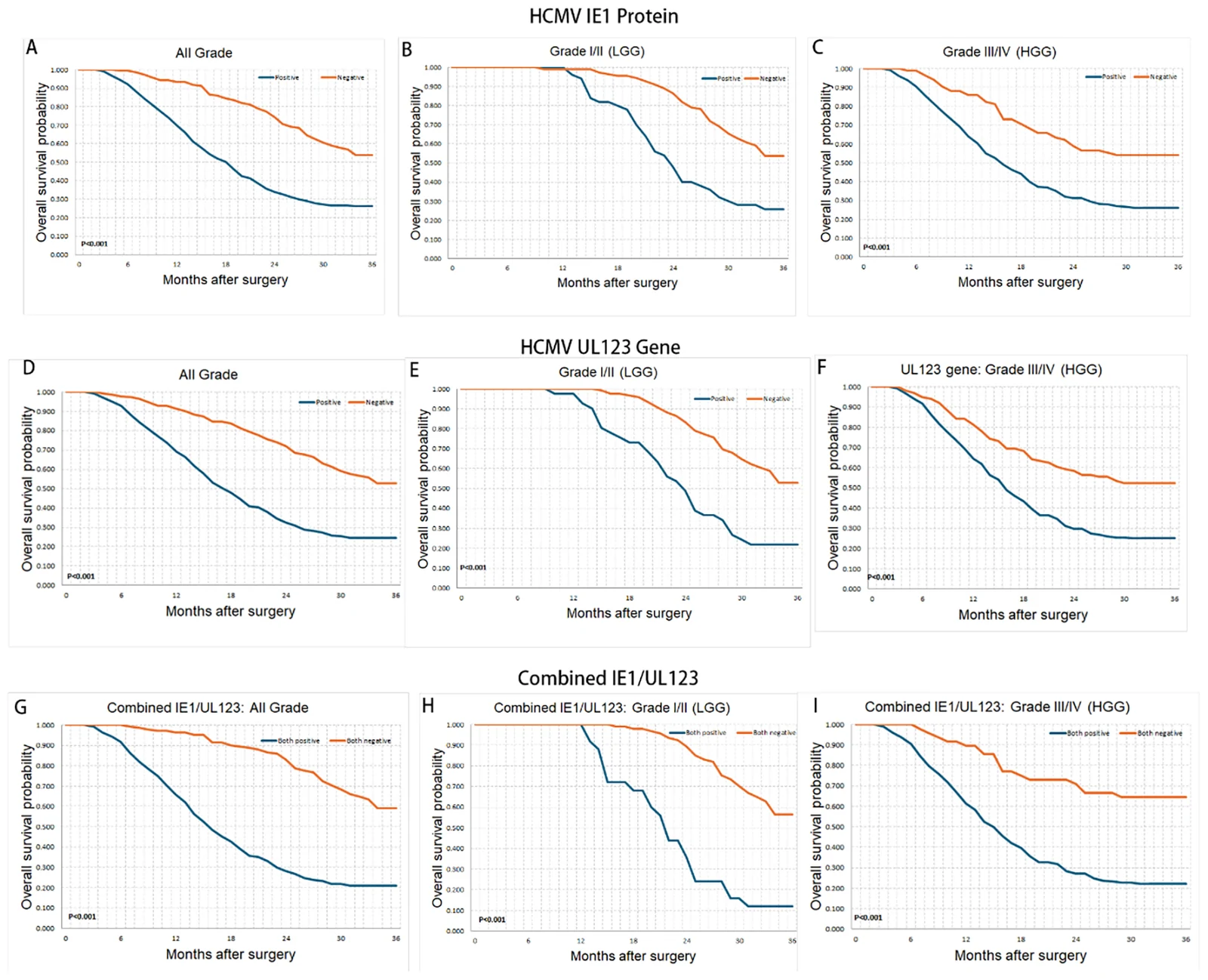
Overall survival according to HCMV IE1 protein and *UL123* DNA status. Kaplan–Meier curves compare overall survival between marker-positive and marker-negative groups. **(A–C)** IE1 protein status in all patients, WHO grade I–II glioma, and WHO grade III–IV glioma, respectively. **(D–F)** *UL123* DNA status in the corresponding groups. **(G–I)** Combined IE1/*UL123* status comparing double-positive and double-negative tumors; discordant cases were excluded.

**Table 4:** Analysis of overall survival according to HCMV marker status.

Marker	WHO grade	Positive n	36-month OS (%)	Median OS (months)	Negative n	36-month OS (%)	Median OS (months)	Log-rank chi-square	P value
IE1 protein	Overall	135	25.9	18.2	86	54.7	NR	26.918	<0.001
IE1 protein	Grade I/II (LGG)	22	22.7	21.5	48	54.2	NR	12.512	<0.001
IE1 protein	Grade III/IV (HGG)	113	26.5	15.8	38	55.3	NR	9.902	0.002
UL123 DNA	Overall	124	25.0	16.7	97	52.6	NR	28.305	<0.001
UL123 DNA	Grade I/II (LGG)	18	22.2	21.3	52	51.9	NR	11.604	<0.001
UL123 DNA	Grade III/IV (HGG)	106	25.5	15.8	45	53.3	NR	10.400	0.001
IE1 protein/UL123 DNA	Overall	100	21.0	15.6	62	59.7	NR	36.866	<0.001
IE1 protein/UL123 DNA	Grade I/II (LGG)	11	9.1	21.3	41	56.1	NR	23.893	<0.001
IE1 protein/UL123 DNA	Grade III/IV (HGG)	89	22.5	15.2	21	66.7	NR	11.788	<0.001

Survival probabilities were estimated using the Kaplan–Meier method and compared using two-sided log-rank tests. Patients with discordant IHC and qPCR results were excluded from the combined-marker analysis. HGG, high-grade glioma; LGG, low-grade glioma; NR, not reached; OS, overall survival.

Kaplan-Meier analysis also showed shorter overall survival in patients with *UL123*-positive tumors than in patients with *UL123*-negative tumors (P<0.001**;** **Figure 2D**). Among the 124 patients with *UL123*-positive tumors, the 36-month overall survival rate was 25.0% and the median overall survival was 16.7 months. Among the 97 patients with *UL123*-negative tumors, the 36-month overall survival rate was 52.6%, and the median overall survival was not reached. Analysis of combined IHC and qPCR results included 100 patients with tumors positive for both IE1 protein and *UL123* DNA and 62 patients with tumors negative for both markers. The 59 patients with discordant results were excluded from this analysis. Overall survival was significantly shorter in the double-positive group than in the double-negative group (P<0.001; **Figure 2G**). The 36-month overall survival rates were 21.0% and 59.7%, respectively, and median overall survival was 15.6 months in the double-positive group but was not reached in the double-negative group.

Survival was further analyzed by WHO tumor grade. Among patients with grade I/II glioma, the 36-month overall survival rate was 22.7% in the IE1-high group (n=22) and 54.2% in the IE1-low group (n=48). Median overall survival was 21.5 months in the IE1-high group and was not reached in the IE1-low group (P<0.001; **Figure 2B**). In patients with grade III/IV glioma, the 36-month overall survival rates were 26.5% for IE1-highhihigh tumors (n=113) and 55.3% for IE1-low tumors (n=38). Median overall survival was 15.8 months in the IE1-high group and was not reached in the IE1-low group (P = 0.002; **Figure 2C**).

A significant difference in overall survival according to *UL123* DNA status was also observed in both WHO grade groups. In grade I/II glioma, the 36-month overall survival rate was 22.2% in the *UL123*-positive group (n=18) and 51.9% in the *UL123*-negative group (n=52). Median overall survival was 21.3 months in the positive group and was not reached in the negative group (P<0.001; **Figure 2E**). In grade III/IV glioma, the 36-month overall survival rates were 25.5% and 53.3% in the *UL123*-positive (n=106) and *UL123*-negative groups (n=45), respectively. Median overall survival was 15.8 months in the positive group and was not reached in the negative group (P = 0.001; **Figure 2F**).

The combined IE1 and *UL123* results remained associated with overall survival in the grade-stratified analyses. In grade I/II glioma, patients with double-positive tumors (n=11) had a 36-month overall survival rate of 9.1% and a median overall survival of 21.3 months. In comparison, the 36-month overall survival rate was 56.1% in patients with double-negative tumors (n=41), and median overall survival was not reached (P<0.001; **Figure 2H**). In grade III/IV glioma, 36-month overall survival was 22.5% in the double-positive group (n=89) and 66.7% in the double-negative group (n=21). Median overall survival was 15.2 months in the double-positive group and was not reached in the double-negative group (P<0.001; **Figure 2I**). In both WHO grade groups, patients with tumors positive for both IE1 protein and *UL123* DNA had shorter overall survival than patients with tumors negative for both markers.

Comparisons were performed using two-sided log-rank tests. The corresponding P values are presented in **Table 4**. HGG, high-grade glioma; LGG, low-grade glioma; OS, overall survival.

## 4 Discussion

This study examined HCMV IE1 protein and UL123 DNA in 221 glioma samples. Both markers were more common in high-grade tumors and were associated with IDH1 wild-type status, older age, lower KPS, and shorter OS. The two tests agreed in most samples, although 59 samples gave different results. These findings support an association between HCMV markers and aggressive glioma features. They do not show that HCMV causes glioma or directly shortens survival.

IE1 protein was detected in 61.1% of samples, whereas UL123 DNA was detected in 56.1%. These rates are close to those reported in several earlier studies and recent reviews (Cobbs et al., 2002; Mitchell et al., 2008; Scheurer et al., 2008; Peredo-Harvey et al., 2021; Moraes et al., 2025). However, other studies found little or no HCMV signal in glioma tissue (Yamashita et al., 2014; Holdhoff et al., 2017). Differences in tissue handling, assay methods, antibodies, DNA targets, and cutoff values may explain part of this variation (Yamashita et al., 2014; Holdhoff et al., 2017; Peredo-Harvey et al., 2021; Moraes et al., 2025). The present results therefore add to the positive evidence but do not resolve the wider debate.

Agreement between IHC and qPCR was 73.3%, with a kappa value of 0.451. This level of agreement shows that the assays are related but cannot be used as substitutes. IHC identified more positive samples than qPCR. A protein signal may remain detectable when viral DNA is below the qPCR limit. Uneven marker distribution within a tumor may also produce different results from adjacent tissue samples. These explanations are possible but were not tested in this study (Peredo-Harvey et al., 2021; Moraes et al., 2025).

HCMV marker positivity was also associated with WHO grade, IDH1 status, age, and KPS. Each of these factors is linked to outcome in glioma. IDH mutations are generally associated with a better prognosis, while older age and poor performance status predict shorter survival (Mirimanoff et al., 2006; Yan et al., 2009). The observed survival differences may therefore reflect differences in these established prognostic factors rather than an independent association with HCMV marker status. Neither marker was associated with sex.

Patients with IE1 protein or UL123 DNA had shorter OS in the Kaplan–Meier analyses. The same pattern was present within low-grade and high-grade groups. It was strongest in patients who were positive by both assays. These results are consistent with earlier reports linking higher HCMV levels to shorter survival (Rahbar et al., 2013; Fornara et al., 2016). However, the present analyses did not include multivariable Cox regression. Hazard ratios and confidence intervals were also not reported. The markers should therefore not be described as independent prognostic factors. The combined analysis excluded 59 patients with different assay results. Its estimates therefore apply only to patients with matching results. Stratification by WHO grade does not account for differences in age, IDH1 status, KPS, or postoperative management.

Several biological studies suggest that HCMV proteins can change signaling, immune responses, and cell behavior (Paulus and Nevels, 2009; Dziurzynski et al., 2012; Fornara et al., 2016). Such effects could help explain the association with more aggressive tumors. However, this study did not assess viral activity, infection timing, or the affected cell type. It also did not test any biological pathway. More aggressive tumors may provide conditions that make HCMV markers easier to detect. HCMV markers may also follow grade or IDH status without having a direct effect. These alternatives remain possible.

Important strengths include the large sample size and paired assessment of protein and DNA in the same tumors. The analysis also compared both markers with several clinical and molecular features. Several limitations should be considered. The findings came from one cohort and require confirmation in independent samples. Assay thresholds and tissue selection may have affected the detection rates. Current WHO classification uses integrated molecular diagnoses, but this study grouped tumors mainly by histologic grade (Louis et al., 2021). IDH1 R132H staining does not identify every IDH1 or IDH2 mutation. Postoperative treatment and extent of resection were not included in the reported survival analyses, and multivariable survival models were not available. Differences in postoperative treatment and extent of resection may therefore contribute to the observed survival associations. The present analyses do not establish whether HCMV marker status provides prognostic information independent of these factors. Differences in postoperative management may therefore contribute to the observed survival associations. The Ct cutoff was not analytically validated, and incomplete control documentation limits interpretation of UL123-negative results.

Conflicting findings have also been reported in other cancers. Harkins et al. detected HCMV nucleic acids and proteins in colorectal neoplasia (Harkins et al., 2002), whereas Bender et al. detected viral DNA in only a subset of colorectal specimens and did not establish a direct association with colorectal cancer. Krishnamoorthy et al. identified HCMV sequences in gastric cancer RNA-seq datasets and used independent tissue testing for confirmation (Krishnamoorthy et al., 2024). In contrast, Johnson et al. found no conclusive evidence of full HCMV transcripts in single-cell RNA-seq data from glioblastoma tumors and cell lines (Johnson et al., 2017). Next-generation sequencing may therefore complement targeted assays, but sequencing findings require independent confirmation and should not be taken as evidence of a causal role in glioma.

Future studies should use standardized, blinded assays and predefined cutoff values. Testing another viral DNA target and sampling several tumor regions could help explain different IHC and qPCR results. Tumors should be classified with current WHO criteria and complete IDH testing (Louis et al., 2021). Survival analyses should report hazard ratios, confidence intervals, and adjusted models, following REMARK guidance (McShane et al., 2005). Independent validation is needed before either marker is used for prognosis. In summary, IE1 protein and UL123 DNA were common in this glioma cohort and were associated with worse clinical features and shorter OS. Future prospective studies using standardized assays, integrated molecular classification, and adjusted survival models may clarify the potential prognostic relevance of HCMV markers in glioma.

## Data Availability

The original contributions presented in the study are included in the article/supplementary material. Further inquiries can be directed to the corresponding author.
